# Trends in long-term opioid prescribing in primary care patients with musculoskeletal conditions: an observational database study

**DOI:** 10.1097/j.pain.0000000000000557

**Published:** 2016-03-18

**Authors:** John Bedson, Ying Chen, Richard A. Hayward, Julie Ashworth, Kate Walters, Kate M. Dunn, Kelvin P. Jordan

**Affiliations:** a Institute for Primary Care and Health Sciences, Staffordshire, United Kingdom; b Research Department of Primary Care and Population Health, UCL, London, United Kingdom

**Keywords:** Analgesic, Opioid, Trends, Prescriptions, Musculoskeletal pain, Long-term care

## Abstract

Long-term opioids may benefit patients with chronic pain but have also been linked to harmful outcomes. In the United Kingdom, the predominant source of opioids is primary care prescription. The objective was to examine changes in the incidence, length, and opioid potency of long-term prescribing episodes for musculoskeletal conditions in UK primary care (2002-2013). This was an observational database study (Clinical Practice Research Datalink, 190 practices). Participants (≥18 years) were prescribed an opioid for a musculoskeletal condition (no opioid prescribed in previous 6 months), and issued ≥2 opioid prescriptions within 90 days (long-term episode). Opioids were divided into short- and long-acting noncontrolled and controlled drugs. Annual incidence of long-term opioid episodes was determined, and for those still in a long-term episode, the percentage of patients prescribed each type 1 to 2 years, and >2 years after initiation. Annual denominator population varied from 1.25 to 1.38 m. A total of 76,416 patients started 1 long-term episode. Annual long-term episode incidence increased (2002-2009) by 38% (42.4-58.3 per 10,000 person-years), remaining stable to 2011, then decreasing slightly to 55.8/10,000 (2013). Patients prescribed long-acting controlled opioids within the first 90 days of long-term use increased from 2002 to 2013 (2.3%-9.9%). In those still in a long-term opioid episode (>2 years), long-acting controlled opioid prescribing increased from 3.5% to 22.6%. This study has uniquely shown an increase in prescribing long-term opioids to 2009, gradually decreasing from 2011 in the United Kingdom. The trend was towards increased prescribing of controlled long-acting opioids and earlier use. Further research into the risks and benefits of opioids is required.

## Abstract 2

Supplemental Digital Content is Available in the Text.

Long-term opioid use decreased from 2011, but the proportion of more potent opioids prescribed increased. Ongoing review of effectiveness and need for discontinuation is important.

## 1. Introduction

Over 20% of adults in the United Kingdom present to primary care with a musculoskeletal (MSK) condition each year.^21^ Guidance from the World Health Organisation and the UK National Institute for Health and Care Excellence (NICE) suggest using opioids as part of a stepped approach to controlling MSK pain.^16,30^ This advice advocates incremental increases from analgesics such as paracetamol to stronger analgesics such as opioids. Previous studies suggest that approximately 50% of patients consulting with MSK pain will be prescribed an analgesic at first consultation,^10,27^ of which 29% will be prescribed an opioid within 2 weeks.^29^

There is evidence that opioid use often continues long-term (>3 months).^3,43^ In some patients with chronic pain, opioid use has been found to be beneficial,^12,31^ but their use has been associated with potential harms including dependence, addiction, self-poisoning, and bone fractures.^9,37^ Increases in prevalent long-term opioid use between 1997 and 2005 have been highlighted in the United States, doubling to approximately 46 per 1000 individuals.^3^ In the United States, the use of opioids is under increasing scrutiny, and governmental measures have been enacted to deal with what is seen as an increasing epidemic of opioid use and abuse.^32^ However, the trends in long-term opioid use seen in the United States may not be reflected in other westernised countries because of differences in health care systems and prescribing guidelines. Two studies have shown substantial increases in UK primary care prescribing of potent opioids from 2001 to 2010. One using a regional (North Staffordshire) general practice database (Consultations in Primary Care Archive, CiPCA) found a doubling of potent opioid use (from 545 to 1032 per 10,000 registered population),^1^ whereas a study using the national Clinical Practice Research Datalink (CPRD), found an increase in prevalence of potent opioid use from 1.8 to 9.2 per 1000 registered patients.^46^ However, none of these studies examined the changing patterns of new primary care prescribing of long-term opioids, and it remains unclear whether the frequency of administering patients long-term opioids is changing given the guidance available. In particular it is not known whether doctors are using more opioids of all types for longer periods of time, and whether, in those patients prescribed long-term opioids, there is a trend in the opioid formulations used towards increasingly potent types. Less potent, noncontrolled opioids can be bought over-the-counter, and opioids are prescribed in secondary care settings such as pain clinics or specialist opioid addiction clinics, but the predominant source of opioid prescriptions is primary care.^7,35^ Understanding what is happening with respect to prescribing long-term opioids in primary care is important because the potential adverse effects and abuse that have been seen in the United States.^26^

The objectives were to examine changes in the incidence and length of episodes of long-term opioid prescribing for MSK conditions in primary care from 2002 to 2013, and assess whether the strength of opioids prescribed to long-term users has changed over that time.

## 2. Methods

This was an observational database study performed in the CPRD, a high-quality anonymised database of routinely recorded information from general practices trained and assessed in their recording of information. The data comprise approximately 14 million patients with around 5.4 million of these being currently active, and registered in 660 primary care practices spread throughout the United Kingdom.^20,40^ In England, 98% of the population are registered with a general practice,^18,33^ and it is in primary care that 90% of all National Health Service (NHS) contacts occur.^15^ The practices that constitute CPRD offer a representative sample of the UK population providing a comprehensive database of primary care prescribing, consultations, and diagnostic coding that has been validated in previous studies.^20,45^ For the purposes of future analysis, practices included were required to have linked Office for National Statistics, deprivation, and Hospital Episode Statistics data. Practices with linkage have been shown to be similar to CPRD practices without linkage on age, deprivation, body mass index, years of follow-up, and prescription of drugs.^13^ We also restricted this analysis to the 190 practices who continuously contributed to CPRD between 2002 and 2013. The denominator population was taken from currently active patients aged 18 and over in these 190 practices.

We identified patients aged 18 years plus, starting a long-term opioid (defined below) at the time of a recorded noninflammatory potentially painful MSK condition in primary care. Visits to the primary care physician for MSK conditions were identified using previously defined Read codes taken from Chapters 1 (History and symptoms), N (Musculoskeletal), R (Symptoms), and S (Injury).^19,21^ Read codes are a commonly used method of recording morbidity in UK primary care. The codes used in this study are available from the authors. Patients were included if the MSK visit occurred within a period lasting from 14 days before the initial opioid prescription, and up to 90 days after it. This was done to link the MSK problem to the prescription, because the opioid prescriptions considered were long term and a painful MSK problem occurring within that time frame will be affected by it. Each participant was required to have at least 12 months of records in the CPRD database before the initial opioid prescription and have no record of cancer before the initial opioid prescription and up to 6 months after the initial opioid prescription.

Opioids were defined as analgesics used to relieve moderate to severe pain from sections 4.7.1 and 4.7.2 of the British National Formulary.^2^ The start of a long-term prescription of opioids was determined as the date of issue of an opioid prescription where the patient had not received any opioid prescription for at least a period of 6 months before this date. An episode of long-term use was defined as at least 3 opioid prescriptions issued within a 90-day period from the date of the new opioid prescription. An episode of long-term opioid use ended when there was a gap of 6 months or more without an opioid prescription. This definition is based on a classification of long-term opioid use that has been used in previous studies.^9,43^ The end date of a long-term opioid episode was set at 28 days after the issue of the last prescription for the opioid in keeping with local health authority guidance to set a maximum of a 28-day supply of medication per prescription and for schedule 2 and 3 opioids, no longer than 30 days.^8,42^ In the United Kingdom, patients do not have to return to their primary care physician for every repeat prescription of their opioid, and can obtain them on a monthly basis from their medical practice using a doctor-authorised repeat prescription service.

Opioids were grouped according to whether they were controlled or noncontrolled drugs. The UK classification of controlled drugs, specifically schedules 2 and 3 of the misuse of drugs legislation,^2,42^ specifies which opioid analgesics are classified as controlled drugs and are subject to specific legal requirements when these drugs are prescribed. Opioids can be compared in potency by using their morphine equivalent dose, which is the amount of morphine that achieves the same analgesic effect of the opioid in question. Controlled drugs are made up of the most potent opioid analgesics such oxycodone, in which 1 mg is equivalent to 1.5 mg of morphine (ie, 50% stronger than morphine). Noncontrolled opioids include less potent opioids such as codeine, in which 1 mg is equivalent to 0.15 mg of morphine. These opioids were then further stratified by determining whether they were a short-acting analgesic (duration of effect 4-6 hours) or a long-acting one (minimum of 12 hours).

### 2.1. Statistical analysis

The total number of patients receiving new episodes of long-term MSK-related opioid prescriptions from 2002 to 2013 was identified and the total number of such episodes as defined above was determined for these patients.

### 2.1.1. Incident prescribing

Annual incidence of episodes of long-term opioid prescribing for MSK conditions was determined for each year from 2002 to 2013. The numerator was all patients starting a new episode of long-term opioids in that calendar year. The denominator population was person-years of all registered patients in a year not currently prescribed opioids. Annual incidence is reported per 10,000 person-years (with 95% confidence interval) stratified by age and sex. For the year 2013, the annual incidence is estimated from the data of the first 9 months because the data for 2014 required to determine new episodes of long-term prescribing for the fourth quarter of 2013 was not available at the time of analysis. We also determined the percentage of episodes lasting more than 1 year, and the percentage lasting more than 2 years, stratified by year of start of episode.

We also determined annual incidence standardised to the age and sex distribution of the England 2013 population. Standardised figures were very similar to unstandardised figures; hence, we report only unstandardised figures in this study.

To determine points of change in trends, we determined the quarterly incidence of long-term opioid prescribing (per 10,000 person-quarters). The quarters were defined on a seasonal basis from the first quarter of 2002 (comprising January, February, and March) to the third quarter of 2013 (July, August, and September). Quarterly incidence figures were seasonally adjusted (using multiplicative model, seasonal decomposition function, SPSS 21). Joinpoint regression was used to identify quarters in which a statistically significant change (the “joinpoint”) in the underlying trend in opioid prescribing occurred.^11,22,28^ The date of these joinpoints can then be matched to dates of external influences such as prescribing guidelines. This does not confirm that the change is linked to these influences but would suggest some influence on prescribing. If no joinpoints are identified, this would indicate no significant change in the underlying trend in prescribing between 2001 and 2013. Permutation tests using Monte Carlo methods were used to determine the minimum number of joinpoints required to provide an adequate fit to the data. The analysis started with the minimum number of joinpoints and tested whether one or more joinpoints were statistically significant (*P* < 0.05) and should be added to the model (up to 5 joinpoints). Models were fitted using the joinpoint regression program (version 4.1.1),^28^ and the best-fitting model was chosen.

### 2.1.2. Subgroup prescribing

We determined the percentage of people prescribed each type of opioid (long or short-acting, controlled, or noncontrolled) at the initial opioid prescription and within 90 days of the start of the episode. Likewise, we determined for those still in a long-term opioid episode the percentage of patients prescribed each type of opioids 1 to 2 years, and more than 2 years after their episode start. If a patient was prescribed more than 1 type of opioid, we used the highest ranking opioid based on the following order (highest to lowest): controlled long-acting opioid (group 4), controlled short-acting opioid (group 3), noncontrolled long-acting opioid (group 2), noncontrolled short-acting opioid (group 1).

### 2.1.3. Sensitivity analysis

A sensitivity analysis was performed also including practices which joined or left CPRD between 2002 and 2013 to determine whether changes in the practice profiles influenced the findings. Analysis was performed using STATA MP 13.1 and SPSS 21.

## 3. Results

**Figure 1.**
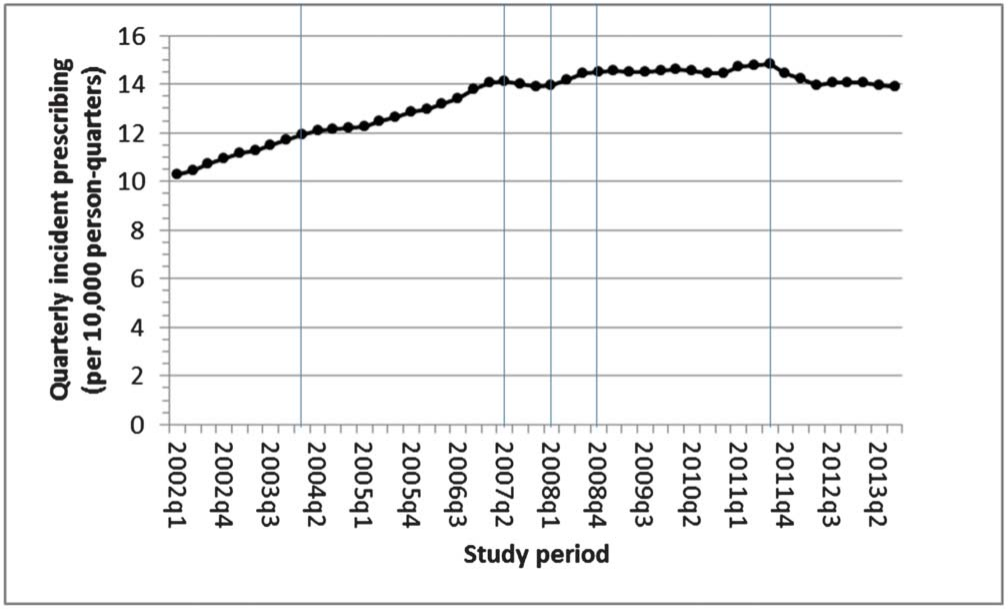
Quarterly incident prescribing of long-term musculoskeletal-related opioids. The lines show the 5 significant (*P* value < 0.05) joinpoints determined in the best-fitting model.

The denominator population varied from 1,253,300 in 2002 to 1,379,217 in 2011, but the age and sex structure remained largely consistent (Appendix 1, available online as Supplemental Digital Content at http://links.lww.com/PAIN/A255). Between 2002 and 2013, a total of 76,416 patients with a potentially painful MSK consultation were identified as starting at least 1 episode of a long-term opioid. In total, 84,184 episodes were identified, with 57,171 (67.9%) being completed episodes by the end of 2013. Uncompleted episodes were either due to end of patient's registration or end of study follow-up. The median episode length was 227 (interquartile range 98, 673) days. This will be an underestimate as it includes the 32% of episodes that were not completed. The annual incidence of long-term opioids for patients with MSK conditions increased by 38% from 2002 (incidence 42.4 per 10,000 person-years) to 2009 (58.3/10,000), and then remained approximately stable until decreasing slightly in 2012 and 2013 to levels that were just below those in 2009 (55.8/10,000) (Table 1). Joinpoint regression analysis indicated the levelling out of the increasing trend started in the fourth quarter of 2008, and the slight decrease in incidence started from the third quarter of 2011 (Fig. 1).

**Table 1.**
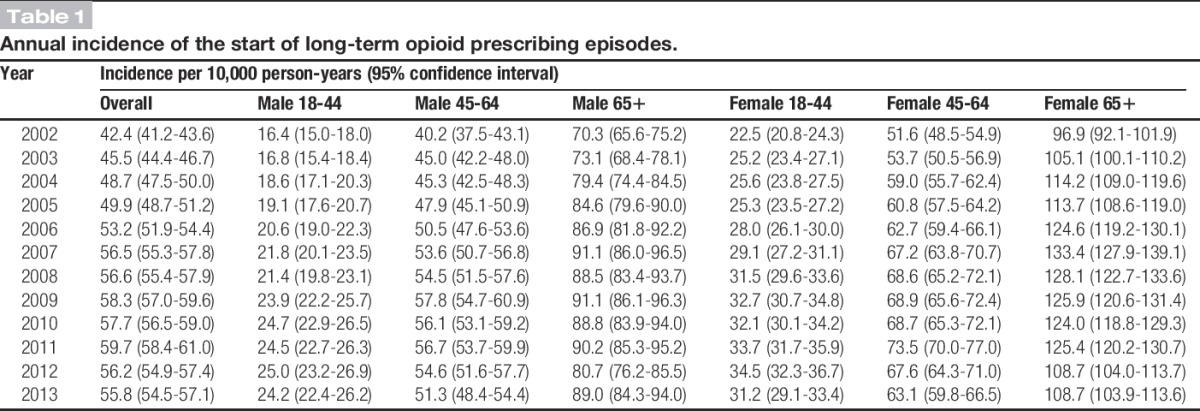
Annual incidence of the start of long-term opioid prescribing episodes.

Females and those in the older age group (≥65 years) were more likely to start new episodes of long-term opioids, but trends over time were similar across sex and age groups (Table 1). Of note, 42.3% of those starting a new long-term opioid prescription in 2002 were in that episode for more than a year, decreasing to 38.8% by 2012, and 28.6% for more than 2 years, which remained consistent over time (Table 2).

**Table 2.**
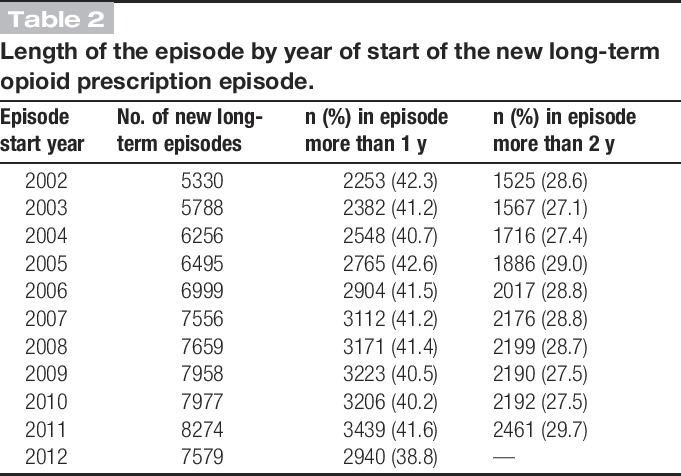
Length of the episode by year of start of the new long-term opioid prescription episode.

### 3.1. Subgroup prescribing

**Figure 2.**
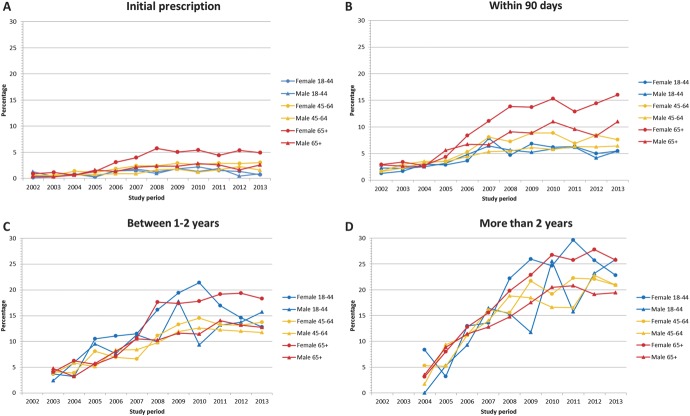
Percentage of patients receiving long-acting controlled opioid prescription by age, sex, and length of time in episode: (A) initial opioid prescription, (B) prescribed within 90 days, (C) using opioids for between 1 - 2 years, and (D) using opioids for more than 2 years.

In 2002, 96.9% of all first prescribed opioids at the start of a long-term opioid episode were short-acting less potent noncontrolled opioids. This decreased slightly to 95.7% by 2013 with the percentage of prescribed long-acting more potent controlled opioids at their initial prescription increasing from 0.7% to 2.8% (Table 3). Between 2002 and 2013, the percentage of people who received a controlled long-term opioid within the first 90 days of their long-term episode increased from 2.3% to 9.9%. By 2013, 14.7% of people who had been in an episode between 1 and 2 years were receiving long-acting more potent controlled opioids, compared with only 4.0% in 2003; 22.6% of patients in a long-term episode for more than 2 years were being prescribed long-acting more potent controlled opioids in 2013, compared with 3.5% in 2004. Females aged 65 and above were prescribed more long-acting potent controlled opioids within 90 days of initial start of a new episode (Fig. 2), but there was little variation by age if episodes lasted more than 1 year (Fig. 2).

**Table 3.**
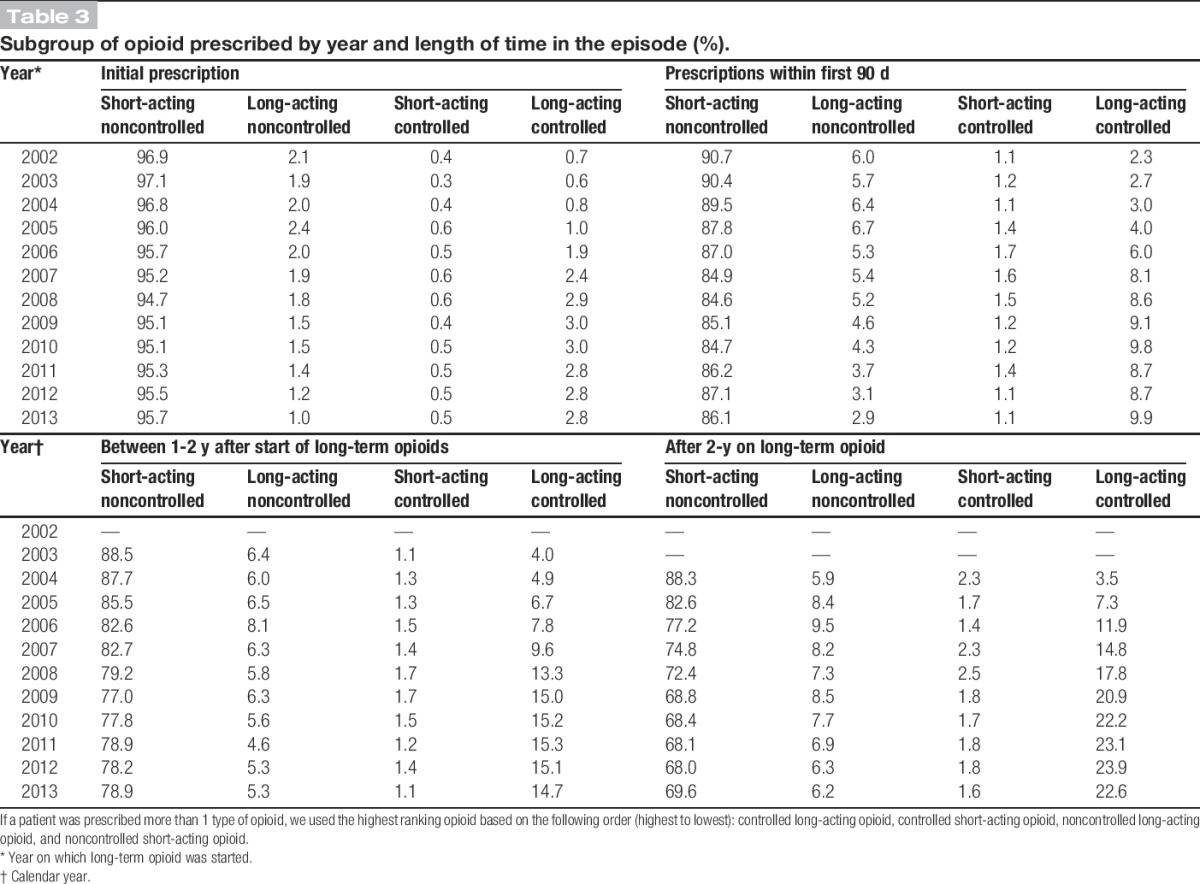
Subgroup of opioid prescribed by year and length of time in the episode (%).

### 3.2. Sensitivity analysis

Including practices that joined or left CPRD during the study period (n = 360) in the analysis gave similar trends to the analysis restricted to those practices continuously contributing to CPRD.

## 4. Discussion

To our knowledge, this is the first study outside the United States to demonstrate an increase specifically in prescribing long-term opioids from 2002 to 2009. However, uniquely, we also observed a smaller gradual decrease between 2011 and 2013 to levels similar to those in 2009. Over the 11 years of the study, there was increased prescribing of the more potent controlled and long-acting, long-term opioids. Additionally, for the first time, we have found that there was also an increase in use of more potent controlled and longer-acting opioids early on in a patient's long-term episode, and over a fifth of patients who had been on opioids for more than 2 years were being prescribed more potent controlled long-acting opioids by 2013.

The increase in incidence of long-term opioids between 2000 and 2009 matches the increasing trend in the prevalence of opioid use among non-cancer pain patients with chronic pain in the United States between 1997 and 2006,^3,34^ and in more potent controlled opioids in the United Kingdom,^46^ although these studies were not restricted to patients with potentially painful MSK conditions. Chevalier et al. showed that from 2006 to 2011, 1 in 5 UK opioid users were prescribed opioids chronically (>183 days of continuous use), and that over the period of their chronic use, the amount they used decreased, but they did not examine in detail trends over time in prescribing and looked at the whole population rather than specifically patients with painful MSK conditions as in this study.^5^ The increased use of long-term opioids may reflect beneficial effects in terms of helping patients with their long-term painful condition, for there is some evidence that they can help in some patients.^12,31^ Indeed, guidelines suggest using opioids if alternative prescribing strategies have failed, which might prompt doctors to use opioids in patients in whom pain has been difficult to control.^16,30^ However, the increasing incidence of prescribing long-term opioids started slowing down shortly after the introduction of the NICE guidance on management of osteoarthritis (February 2008) when the recommendation was to use opioids only after weaker analgesia had been tried. Shortly after the US Government's Office of National Drug Control Policy guidance (April 2011),^32^ the incidence of long-term opioid prescribing in the United Kingdom started to gradually decline to the same levels as those in 2009. The increased use of opioids over the previous decade in the United States and an apparent increase in abuse of long-acting opioids had led to this US initiative.^26^ Although any direct connection of the US guidance to prescribing in the United Kingdom cannot be determined here, it is possible that its message and the well-documented increase in opioid prescribing in the United States^26^ may have had some “spill over” effect through the medical media into the United Kingdom.

Since 2013, initiatives in the United Kingdom have recommended action to underpin regulations designed to improve monitoring and use of controlled drugs such as strong opioids.^4,6^ However, it would appear from this study that nationally from 2011, primary care physicians had already acted to reduce their use of new opioids, but in cases in which opioids were being prescribed, the shift towards using the more potent controlled and long-acting opioids continued. The increased use of controlled opioids is in keeping with the findings of other European studies.^17,36,46^ Although less potent noncontrolled opioid prescriptions still make up the majority of initial prescriptions for opioids, it was evident that primary care physicians did change to the more potent controlled opioids even within the first 90 days of a long-term episode. Those on more potent controlled opioids, either short-acting or long-acting, are of the greatest concern in relation to prescription opioid drug abuse. Our results show that a quarter of patients starting long-term opioids will still be on them more than 2 years later, and over a fifth of these will be on long-acting controlled prescriptions, raising concerns about the potential for abuse and addiction among this group of users. Guidelines that are now being incorporated into normal practice^4,6^ need to ensure clear messages around appropriate use of opioids, including the correct dosage and indications for continued use. Regular review would also ensure that opioid use is only continued when necessary, reducing the potential for addiction and side effects.

Those in the older age groups had higher rates of long-term opioid prescribing and of stronger opioids early on in these episodes. A higher rate of opioid use in older age groups has also been found in other studies.^14,36,46^ This group poses a dilemma for clinicians, as they are at increased risk of side effects,^9,37^ and hence the clinically rational approach would be to use less potent opioids in these patients. However, they are more likely to be affected by severely painful MSK conditions,^21^ and are more likely to have adverse effects from alternatives, such as nonsteroidal anti-inflammatory drugs.^23^ Females had higher rates of new prescriptions for long-term opioids compared with males. This is also consistent with other work,^3,34,36^ and may reflect the higher prevalence of painful MSK conditions among females.^21^

This study used a novel method to identify opioid subgroups using the classification according to the UK Misuse of Drugs Act schedule of controlled drugs.^41^ We did not use morphine equivalent dose or daily defined dose^44^ as has been used in previous studies,^17,46^ but this classification allows direct comparison of less potent (noncontrolled) opioids with the more potent controlled opioids of schedules 2 and 3 that are of major concern for prescribers and regulating bodies in terms of the potential for abuse of long-acting opioid medications.^4,6^ Opioids cannot be directly marketed to patients in the United Kingdom, unlike in the United States where marketing may have influenced increases in the use of long-term opioids.^39^ Management of established abuse and addiction is included in UK guidelines, which clearly define the prescribing of pharmacological interventions to treat addiction.^7^ These strict criteria, which often require the use of specialist services, predetermine opioid use and will have some bearing on the opioid prescribing we have described. However, within the opioids examined here, methadone (an opioid primarily used in the United Kingdom as a substitute in opioid withdrawal) was not included as one of the opioids we examined for this reason.

An advantage of our study was that all prescribing is recorded in CPRD at the point of medication issue in the visit to the primary care physicians, and all repeat prescriptions of these drugs are recorded automatically so missing any opioid data is unlikely. We examined only patients who had potentially painful MSK conditions, and our findings may not be generalisable to opioids prescribed for other conditions, such as pain in cancer, or neuropathic pain. However, among patients reporting chronic pain, there are comparatively few with neuropathic pain.^38^ Some errors in underrecording or coding of MSK conditions might have occurred leading to an underestimation of opioid use. However, historically, GPs in practices contributing to CPRD have been required to enter morbidity codes at a minimum for a new diagnosis or when treatment has changed; therefore, most new prescribing of opioids in consulters should have been identified.^24^ It is possible that not all the opioids within an episode were prescribed for the initial MSK condition.

This analysis relates to information on all patients prescribed opioids, and not just those with health insurance as in studies from the United States, thereby making the results more generalisable to westernised populations outside the United States. Accordingly, the increasing use of long-acting stronger opioids in patients on long-term opioids indicates the continuing need to promote vigilance regarding the ongoing use of opioids among patients with MSK conditions in Europe and further afield. Although the overall prescribing of opioids for MSK conditions has reduced in the most recent years, in the light of recent evidence suggesting lack of effectiveness of paracetamol for knee and back pain,^25^ doctors may in the future change their prescribing habits again in an attempt to help resolve their patients' MSK pain, possibly driving a further increase in prescribing opioids. Monitoring with regular review and assessment of analgesic needs in the patients receiving opiates might reduce the potential for their abuse and helping to determine their effectiveness in controlling chronic pain. Further research to determine whether such changes are being implemented will be required in the future.

## Conflict of interest statement

The authors have no conflicts of interest to declare.

## Supplementary Material

SUPPLEMENTARY MATERIAL

## Appendix A Supplemental Digital Content

Supplemental Digital Content associated with this article can be found online at http://links.lww.com/PAIN/A255.

## Supplemental media

Video content associated with this article can be found online at http://links.lww.com/PAIN/A256.

## References

[1] Bedson J Belcher J Martino OI Ndlovu M Rathod T Walters K Dunn KM Jordan KP The effectiveness of national guidance in changing analgesic prescribing in primary care from 2002 to 2009: an observational database study. Eur J Pain 2013;17:434–43. 22865816 10.1002/j.1532-2149.2012.00189.x PMC3592995

[2] BNF. British national formulary. London: BMJ Group, 2014.

[3] Boudreau D Von Korff M Rutter CM Saunders K Ray GT Sullivan MD Campbell CI Merrill JO Silverberg MJ Banta-Green C Weisner C Trends in long-term opioid therapy for chronic non-cancer pain. Pharmacoepidemiol Drug Saf 2009;18:1166–75. 19718704 10.1002/pds.1833 PMC3280087

[4] Care Quality Commission. The safer management of controlled drugs, Annual report 2012. 2013 Available at: http://www.cqc.org.uk/sites/default/files/documents/cdar_2012.pdf. Accessed September 19, 2015.

[5] Chevalier P Smulders M Chavoshi S Sostek M LoCasale R A description of clinical characteristics and treatment patterns observed within prescribed opioid users in Germany and the UK. Pain Manag 2014;4:267–76. 25300384 10.2217/pmt.14.26

[6] Department of Health. Controlled drugs (supervision of management and use) regulations 2013. 2013 Available at: https://www.gov.uk/government/uploads/system/uploads/attachment_data/file/214915/15-02-2013-controlled-drugs-regulation-information.pdf. Accessed September 19, 2015.

[7] Department of Health (England) and the Devolved Administrations. Drug misuse and dependence: UK guidelines on clinical management. London: Department of Health (England), the Scottish Government, Welsh Assembly Government and Northern Ireland Executive, 2007.

[8] Dowd A Some PCTs recommend GPs limit prescriptions to 28 days. BMJ 2011;342:d2410.

[9] Dunn KM Saunders KW Rutter CM Banta-Green CJ Merrill JO Sullivan MD Weisner CM Silverberg MJ Campbell CI Psaty BM Von Korff M Opioid prescriptions for chronic pain and overdose. Ann Intern Med 2010;152:85–92. 20083827 10.1059/0003-4819-152-2-201001190-00006 PMC3000551

[10] Edwards JJ Jordan KP Peat G Bedson J Croft PR Hay EM Dziedzic KS Quality of care for OA: the effect of a point-of-care consultation recording template. Oxford: Anonymous Rheumatology, 2014. 10.1093/rheumatology/keu411 PMC4416084 25336538

[11] Fay MP Tiwari RC Feuer EJ Zou Z Estimating the average annual percent change for disease rates without assuming constant change. Biometrics 2006;62:847–54. 16984328 10.1111/j.1541-0420.2006.00528.x

[12] Furlan AD Sandoval JA Mailis-Gagnon A Tunks E Opioids for chronic noncancer pain: a meta-analysis of effectiveness and side effects. CMAJ 2006;174:1589–94. 16717269 10.1503/cmaj.051528 PMC1459894

[13] Gallagher AM Puri S Van Staa T Linkage of the General Practice Research Database (GPRD) with other data sources. Pharmacoepidemiol Drug Saf 2011;20:S230–231.

[14] Green DJ Bedson J Blagojevic-Burwell M Jordan KP van der Windt D Factors associated with primary care prescription of opioids for joint pain. Eur J Pain 2013;17:234–44. 22718522 10.1002/j.1532-2149.2012.00185.x

[15] Gregory S General practice in England: an overview. The King's Fund, 2009 Available at: http://www.kingsfund.org.uk/sites/files/kf/general-practice-in-england-overview-sarah-gregory-kings-fund-september-2009.pdf. Accessed November 27, 2015.

[16] Gureje O Von Korff M Simon GE Gater R Persistent pain and well-being: a World Health Organization Study in Primary Care. JAMA 1998;280:147–51. 9669787 10.1001/jama.280.2.147

[17] Hamunen K Paakkari P Kalso E Trends in opioid consumption in the Nordic countries 2002–2006. Eur J Pain 2009;13:954–62. 19091608 10.1016/j.ejpain.2008.11.006

[18] Health and Social Care Information Centre. Numbers of patients registered at a GP practice—April 2014. 2014 Available at: http://www.hscic.gov.uk/catalogue/PUB13932. Accessed November 27, 2015.

[19] Health and Social Care Information Centre. Read codes. 2015 Available at: http://systems.hscic.gov.uk/data/uktc/readcodes. Accessed September 19, 2015.

[20] Herrett E Thomas SL Schoonen WM Smeeth L Hall AJ Validation and validity of diagnoses in the General Practice Research Database: a systematic review. Br J Clin Pharmacol 2009;69:4–14. 20078607 10.1111/j.1365-2125.2009.03537.x PMC2805870

[21] Jordan K Kadam U Hayward R Porcheret M Young C Croft P Annual consultation prevalence of regional musculoskeletal problems in primary care. BMC Musculoskelet Disord 2010;11:144. 20598124 10.1186/1471-2474-11-144 PMC2903510

[22] Kim HJ Fay MP Feuer EJ Midthune DN Permutation tests for joinpoint regression with applications to cancer rates. Stat Med 2000;19:335–51. 10649300 10.1002/(sici)1097-0258(20000215)19:3<335::aid-sim336>3.0.co;2-z

[23] Lanza F Chan F Quigley E Guidelines for prevention of NSAID-related ulcer complications. Am J Gastroenterol 2009;104:728–38. 19240698 10.1038/ajg.2009.115

[24] Lawson D Sherman V Hollowell J The general practice research database. Q J Med 1998;91:445–52. 10.1093/qjmed/91.6.445 9709463

[25] Machado GC Maher CG Ferreira PH Pinheiro MB Lin CWC Day RO McLachlan AJ Ferreira ML Efficacy and safety of paracetamol for spinal pain and osteoarthritis: systematic review and meta-analysis of randomised placebo controlled trials. BMJ 2015;350:h1225. 25828856 10.1136/bmj.h1225 PMC4381278

[26] Manchikanti L Fellows B Ailinani H Pamapti P Therapeutic use, abuse, and nonmedical use of opioids: a ten-year perspective. Pain Phys 2010;13:401–35. 20859312

[27] Muller S Bedson J Mallen CD The association between pain intensity and the prescription of analgesics and non-steroidal anti-inflammatory drugs. Eur J Pain 2012;16:1014–20. 22337613 10.1002/j.1532-2149.2011.00107.x PMC3564413

[28] National Cancer Institute. Joinpoint regression program. 2015 Available at: http://surveillance.cancer.gov/joinpoint/. Accessed September 19, 2015.

[29] Ndlovu M Bedson J Jones PW Jordan KP Pain medication management of musculoskeletal conditions at first presentation in primary care: analysis of routinely collected medical record data. BMC Musculoskelet Disord 2014;15:1–11. 25492581 10.1186/1471-2474-15-418 PMC4295256

[30] NICE. Osteoarthritis care and management in adults: NICE clinical guideline 177. 2014 Available at: http://www.nice.org.uk/nicemedia/live/14383/66527/66527.pdf. Accessed September 16, 2015.

[31] Noble M Treadwell JR Tregear SJ Coates VH Wiffen PJ Akafomo C Schoelles KM Long-term opioid management for chronic noncancer pain. Cochrane Database Syst Rev 2010;1:CD006605. 20091598 10.1002/14651858.CD006605.pub2 PMC6494200

[32] Office of National Drug Control Policy. Epidemic: responding to America's prescription drug abuse crisis. 2011 Available at: http://www.whitehouse.gov/sites/default/files/ondcp/issues-content/prescription-drugs/rx_abuse_plan.pdf. Accessed September 19, 2015.

[33] ONS. Mid-year population estimates for the UK 2014. 2015 Available at: http://www.ons.gov.uk/ons/rel/pop-estimate/population-estimates-for-uk–england-and-wales–scotland-and-northern-ireland/mid-2014/mid-year-population-estimates-for-the-uk-2014.html. Accessed November 27, 2015.

[34] Parsells Kelly J Cook SF Kaufman DW Anderson T Rosenberg L Mitchell AA Prevalence and characteristics of opioid use in the US adult population. PAIN 2008;138:507–13. 18342447 10.1016/j.pain.2008.01.027

[35] Prescribing and Medicines Team Health and Social Care Information Centre. Prescriptions dispensed in the community England 2004–14. 2015 Available at: http://www.hscic.gov.uk/catalogue/PUB17644/pres-disp-com-eng-2004-14-rep.pdf. Accessed April 4, 2016.

[36] Ruscitto A Smith BH Guthrie B Changes in opioid and other analgesic use 1995–2010: repeated cross-sectional analysis of dispensed prescribing for a large geographical population in Scotland. Eur J Pain 2015;19:59–66. 24807782 10.1002/ejp.520

[37] Saunders K Dunn KM Merrill J Sullivan MD Weisner C Brennan Braden J Psaty BM Von Korff M Relationship of opioid use and dosage levels to fractures in older chronic pain patients. J Gen Intern Med 2010;4:310–15. 20049546 10.1007/s11606-009-1218-z PMC2842546

[38] Smith BH Torrance N Epidemiology of neuropathic pain. Pain Manag 2011;1:87–96. 24654588 10.2217/pmt.10.5

[39] Stannard C Opioid prescribing in the UK: can we avert a public health disaster? Br J Pain 2012;6:7–8. 26516459 10.1177/2049463712439131 PMC4590089

[40] Tate AR Beloff N Al-Radwan B Wickson J Puri S Williams T Van Staa T Bleach A Exploiting the potential of large databases of electronic health records for research using rapid search algorithms and an intuitive query interface. J Am Med Inform Assoc 2014;21:292–8. 24272162 10.1136/amiajnl-2013-001847 PMC3932457

[41] The Advisory Council on the Misuse of Drugs. Misuse of drugs act 1971. 1971 Available at: http://www.legislation.gov.uk/ukpga/1971/38/pdfs/ukpga_19710038_en.pdf. Accessed September 19, 2015.

[42] UK Statutory Instruments. The misuse of drugs regulations. 2001 Available at: http://www.legislation.gov.uk/uksi/2001/3998/made. Accessed September 19, 2015.

[43] Von Korff M Saunders K Thomas Ray G Boudreau D Campbell C Merrill J Sullivan MD Rutter CM Silverberg MJ Banta-Green C Weisner C De facto long-term opioid therapy for noncancer pain. Clin J Pain 2008;24:521–7. 18574361 10.1097/AJP.0b013e318169d03b PMC3286630

[44] WHO. Daily defined dose: definition and general considerations. 2014 Available at: http://www.whocc.no/ddd/definition_and_general_considera/. Accessed September 19, 2015.

[45] Williams T van Staa T Puri S Eaton S Recent advances in the utility and use of the General Practice Research Database as an example of a UK Primary Care Data resource. Ther Adv Drug Saf 2012;3:89–99. 25083228 10.1177/2042098611435911 PMC4110844

[46] Zin CS Chen LC Knaggs RD Changes in trends and pattern of strong opioid prescribing in primary care. Eur J Pain 2014;18:1343–51. 24756859 10.1002/j.1532-2149.2014.496.x PMC4238849

